# Combined predictive value of TyG index and PLR for mental health in Chinese adults: A machine learning approach

**DOI:** 10.1371/journal.pone.0355937

**Published:** 2026-09-02

**Authors:** Jianfan Zhou, Shuting Yin, Shuan Xue, Chunhua Sun, Yuan Zhao

**Affiliations:** 1 School of Physical Education, Shandong University, Jingshi Road, Lixia District, Jinan, Shandong, China; 2 College of Health Sciences, Shandong University of Traditional Chinese Medicine, Daxue Road, Changqing District, Jinan, Shandong, China; 3 Department of Health Management Center, Qilu Hospital of Shandong University, 107 Wenhua West Road, Jinan, Shandong, China; The First Affiliated Hospital of Soochow University, CHINA

## Abstract

**Objective:**

To investigate the independent and combined associations of the triglyceride-glucose (TyG) index and platelet-to-lymphocyte ratio (PLR) with mental health in Chinese adults, and to evaluate their predictive value using both traditional regression and machine learning approaches.

**Method:**

Data from 725 adults at Qilu Hospital of Shandong University were analyzed. Mental health was evaluated using Symptom Checklist-90 (SCL-90). Fasting blood samples were used to calculate the TyG index and PLR. Linear and logistic regressions evaluated associations with overall and domain-specific mental health. The predictive performance of the TyG index and PLR was further evaluated using logistic regression and six machine learning classifiers, based on an 80/20 train-test split with cross-validation. Multiple performance metrics—including AUC, sensitivity, specificity, and MCC—were reported, and LASSO regression was applied to identify key predictors.

**Results:**

The TyG index was positively associated with the SCL-90 total score, somatization, interpersonal sensitivity, depression, anxiety, and hostility (P < 0.05); PLR showed similar associations and was also associated with phobic anxiety and psychoticism (P < 0.05). Individuals in the high-TyG/high-PLR group had significantly higher scores across all SCL-90 dimensions (P < 0.05), except for obsessive-compulsive symptoms and paranoid ideation. Among the six machine learning models, LASSO regression demonstrated the best overall predictive performance for mental health problems (AUC = 0.744), showing balanced sensitivity, specificity, F1-score, and MCC. PLR and the TyG index were identified as the strongest positive predictors. Compared with traditional logistic regression, machine learning models showed superior discriminative ability and enabled the assessment of variable importance.

**Conclusions:**

The TyG index and PLR are independently and jointly associated with mental health indicators in Chinese adults. Their combination may enhance the ability to identify individuals at elevated risk for mental health problems and could serve as a useful biomarker pair in predictive applications.

## 1. Introduction

In recent years, the world has been facing an increasingly severe mental health crisis [1]. It is estimated that approximately one billion people globally are afflicted by various forms of mental health issues, and this number continues to rise steadily [2]. Mental health problems not only significantly reduce individuals’ quality of life and social adaptability [3], but are also closely associated with social problems such as decreased work efficiency and family dysfunction. Simultaneously, psychological issues constitute significant risk factors for increased disease burden, elevated disability rates, and premature death [4,5]. Especially in the post-pandemic era, psychological problems like anxiety and depression are showing a high incidence globally, necessitating the urgent exploration of feasible early identification and intervention methods to effectively prevent and control mental health issues.

Insulin resistance (IR) refers to a pathological state where the body’s efficiency in glucose uptake and utilization mediated by endogenous or exogenous insulin is diminished [6]. As a core mechanism of metabolic disorder, it is closely associated with major chronic diseases such as all-cause mortality [7], atherosclerosis [8], cardiovascular diseases [9], and diabetes [10]. Recent studies have also identified associations between IR and cognitive decline, depression, and broader indicators of mental health impairment [11]. Although the hyperinsulinemic-euglycemic clamp remains the gold standard for assessing IR, its high cost and invasiveness limit its feasibility in large-scale population-based research [12]. In this context, the triglyceride-glucose (TyG) index, derived from routine clinical measures, has emerged as a cost-effective and non-invasive surrogate marker for IR, with demonstrated diagnostic agreement [13]. Notably, data from the National Health and Nutrition Examination Survey (NHANES) have shown positive associations between elevated TyG index levels and symptoms of anxiety [14], as well as depression scores [15]. However, most existing evidence primarily comes from Western populations and focuses on a narrow set of psychological outcomes. In Chinese populations, psychological distress often manifests as somatization, hostility, and interpersonal sensitivity, partly due to cultural norms that discourage the direct expression of emotions [16]. Cross-cultural research has also shown that Asians tend to somatize negative experiences to a greater extent than Westerners [17]. Therefore, the potential associations between the TyG index and these culturally shaped psychological dimensions remain insufficiently explored and warrant further investigation.

Beyond metabolic dysregulation, inflammation-an essential pathway through which the immune system responds to internal and external stressors, also plays a critical role in the pathophysiological mechanisms underlying mental health and psychiatric disorders [18,19]. A substantial body of evidence from animal experiments, cohort studies, and systematic reviews has demonstrated that elevated levels of inflammatory markers are robustly associated with depression and other mental health problems [20–22]. The platelet-to-lymphocyte ratio (PLR), a simple and readily obtainable indicator of systemic inflammation [23], has been shown to be related to various chronic diseases as well as psychological symptoms [24,25]. Notably, metabolic abnormalities (reflected by TyG) and inflammatory activation (reflected by PLR) are not independent of each other; rather, they constitute two core biological pathways that jointly shape psychological health. Insulin resistance can induce the upregulation of inflammatory cytokines [26], while persistent inflammation can impair insulin signaling and promote metabolic abnormalities [27], forming a bidirectionally reinforcing pathological cycle. This dual-pathway mechanism is further supported by several integrated theoretical frameworks. Psychoneuroimmunology synthesizes the neural, endocrine, and immune systems, emphasizing the interactive roles of metabolic and inflammatory processes in psychological health [28]. The Allostatic Load model posits that chronic stress accumulates physiological burden through multiple systems, including metabolic and inflammatory pathways, ultimately compromising psychological well-being [29]. Additionally, the inflammatory hypothesis of depression highlights the central role of inflammation in the development of mental disorders [30]. Based on these frameworks, assessing metabolic or inflammatory markers in isolation may underestimate the contribution of the other pathway and fail to capture their interactive and synergistic effects. Therefore, incorporating both TyG index and PLR into the same analytic model is not only theoretically justified but also facilitates a more comprehensive characterization of an individual’s multisystem physiological burden, thereby providing a more accurate understanding of the biological underpinnings of mental health problems.

Moreover, current mental health risk screening relies largely on self-reported questionnaires, which are vulnerable to emotional fluctuations and cognitive biases, thereby introducing subjectivity and limiting their diagnostic accuracy. In contrast, the TyG index and PLR are obtained from routine biochemical tests and therefore offer objective, low-cost, and widely accessible measures—features that are especially valuable in primary care settings or resource-limited regions. If validated, these indicators could serve as practical complements to traditional questionnaire-based screening. Based on this, this study aims to explore the independent and combined associations of TyG index and PLR with mental health, and to evaluate their predictive value for identifying mental health problems. Importantly, associations between metabolic and inflammatory markers and psychological outcomes may be nonlinear, interactive, and multidimensional—features that traditional regression models are often inadequate to capture. To address this complexity, the study incorporates multiple machine learning algorithms to assess the predictive value of TyG index and PLR. Compared with traditional regression, machine learning offers superior capacity for modeling nonlinearities, higher-order interactions, and complex feature structures, thereby providing a more comprehensive evaluation of the contribution of biological indicators to mental health problem. This methodological advancement strengthens the assessment of TyG index and PLR from both predictive and mechanistic perspectives.

## 2. Methods

### 2.1. Participants

Participants for this study were recruited from the Health Management Center of Qilu Hospital, Shandong University, between July 19, 2024, and July 18, 2025. Participants were eligible for inclusion if they voluntarily agreed to take part in the study and provided informed consent to undergo the psychological health screening. Individuals were excluded if they had conditions that could compromise the accuracy of the measurements, such as severe physical or cognitive impairments. This study was conducted using a modular design, with participants voluntarily choosing to participate in each module. Among those with complete blood test data and SCL-90 outcomes, 804 participants were initially eligible. After excluding individuals with missing demographic information or other covariates, a total of 725 participants were included in the final analysis. The study was conducted in accordance with the Declaration of Helsinki and received ethical approval from the Institutional Review Board of Qilu Hospital, Shandong University (Approval No. KYLL-202406-035-1). The ethics committee granted a waiver of informed consent.

### 2.2. Mental health

This study used the Chinese version of the Symptom Checklist-90 (SCL-90) to evaluate the mental health of participants. The SCL-90 consists of 90 items, covering nine dimensions of psychological symptoms: somatization, obsessive-compulsive, interpersonal sensitivity, depression, anxiety, hostility, phobic anxiety, paranoid ideation, and psychoticism. Each item is rated on a 5-point scale (1 = never, 5 = severe), with a higher total score indicating poorer mental health status. To assess the internal consistency of the scale, Cronbach's α coefficient was calculated based on standardized items, resulting in a value of 0.969. This indicates excellent internal consistency reliability among the study participants. According to commonly used criteria, the presence of mental health issues is indicated if the total score exceeds 160, the number of positive items is greater than 43, or any single dimension score exceeds 2 [31].

### 2.3. Blood examination

In this study, all participants underwent fasting venous blood sampling (after a minimum of 8 hours of fasting) between 8:00 and 10:00 AM on the same day. The blood samples were collected by professionally trained nurses and promptly transported to the laboratory for biochemical analysis. The biochemical markers measured included fasting blood glucose (FBG), total cholesterol (TC), triglycerides (TG), high-density lipoprotein cholesterol (HDL-C), low-density lipoprotein cholesterol (LDL-C), platelet count (PLT), and lymphocyte count (LYM). Biochemical assays were performed using an automated biochemical analyzer, and blood routine tests were conducted using an automated hematology analyzer. Based on the test results, the following calculations were further performed: ① The triglyceride-glucose index (TyG index) = ln[TG(mg/dL) × FBG(mg/dL)/2], which serves as an indicator of insulin resistance levels; ② The platelet-to-lymphocyte ratio (PLR) = PLT / LYM, which acts as a potential marker of inflammatory status. Both the neutrophil-to-lymphocyte ratio (NLR) and the PLR are relatively stable biomarkers of systemic inflammation. Previous studies have suggested that PLR may be a better predictor than NLR in assessing the severity of inflammation [32,33]. Therefore, in this study, PLR was chosen to evaluate inflammation levels. All biochemical measurements and calculations were performed using standardized procedures to ensure data accuracy and consistency.

### 2.4. Covariates

The primary confounding factors considered in this study include age, gender, BMI, education level, smoking, alcohol consumption, hypertension, diabetes, and dyslipidemia. Age, gender, and education level (junior high school or below, high school, college degree, college degree or above), smoking status (current smoker: yes or no), and alcohol consumption (currently consumes alcohol: yes or no) were all extracted from the hospital’s electronic medical records. Participants wore light clothing and underwent fasting measurements of height and weight to calculate BMI (underweight, normal weight, overweight, obesity). Systolic blood pressure (SBP) and diastolic blood pressure (DBP) were measured while participants were in a resting state. Hypertension was defined as a self-reported history, SBP ≥ 140 mmHg, or DBP ≥ 90 mmHg. Diabetes was defined as a self-reported history or fasting blood glucose ≥7.0 mmol/L. Dyslipidemia was defined as one or more lipid parameters (HDL-C, LDL-C, TG, TC) being outside the normal range (see Supplementary Table S1 in S1 File for detailed specification).

### 2.5. Data analysis

Continuous variables were presented as mean ± standard deviation, while categorical variables were presented as frequencies and percentages. The Mann-Whitney U test and chi-square test were used to assess differences between groups for continuous and categorical variables, respectively. Participants were categorized into high/low TyG and high/low PLR groups based on median values, resulting in four combinations: low TyG and low PLR, low TyG and high PLR, high TyG and low PLR, and high TyG and high PLR.

For regression analyses, two levels of covariate adjustment were defined. Model 1 (minimally adjusted model) included age and gender. Model 2 (fully adjusted model) included age, gender, BMI, smoking status, alcohol consumption, education level, diabetes, hypertension, and dyslipidemia. Multiple linear regression models were used to examine the independent associations of the TyG index and PLR with the total SCL-90 score and its sub-dimensions. In the independent association analyses, TyG index and PLR were mutually adjusted in Model 2. To evaluate their joint association, both TyG index and PLR were simultaneously included in the regression models without mutual exclusion. Subsequently, multiple logistic regression analyses were performed to examine the independent and joint associations of the TyG index and PLR with psychological health status defined by the SCL-90, using the same covariate adjustment strategy.

To evaluate the discriminatory ability of the TyG index and PLR for identifying participants with psychological health problems, we implemented traditional logistic regression and six machine learning classifiers: LASSO logistic regression, Random Forest (RF), Support Vector Machine (SVM), Gradient Boosting Machine (GBM), XGBoost, and an artificial neural network (ANN) in R. The dataset was split into training (80%) and testing (20%) sets using stratified sampling. To address outcome imbalance, ROSE oversampling was applied to the training set. Continuous predictors (TyG index, PLR, and age) were standardized based on the training data, with identical transformations applied to the testing set. Hyperparameters were tuned via 10-fold cross-validation within the training set, and optimal configurations were determined by grid search (Supplementary Table S2 in S1 File). LASSO regression was implemented with the glmnet package (L1 penalty, α = 1), with the penalty parameter λ selected via cross-validation. RF models (randomForest package) used 1000 trees, with mtry optimized via out-of-bag error. SVMs employed a radial basis function kernel, tuning cost and gamma via grid search. GBM models (gbm package) were tuned for tree number, interaction depth, shrinkage, and minimum observations per node. XGBoost models (xgboost package) used a binary logistic objective, with hyperparameters optimized by 10-fold cross-validation with early stopping. The ANN consisted of an input layer with all predictors, two hidden layers with 8 and 4 neurons, respectively, and a single-node output layer with logistic activation. Training employed backpropagation, with a maximum of 1 × 10⁶ iterations and convergence threshold 0.1, producing probabilistic outputs (linear.output = FALSE). Model performance was evaluated on the independent testing set using AUC, sensitivity, specificity, accuracy, F1-score, Cohen’s Kappa, Brier score, and Matthews correlation coefficient. Predictor importance was assessed via LASSO coefficients, XGBoost Gain, RF variable importance, and GBM relative influence. All analyses were conducted using RStudio and Stata 17.0.

## 3. Results

This study included a total of 725 participants with a mean age of 52.54 years, including 400 males and 325 females. The mean PLR was 139.58, the mean TyG index was 8.58, and the mean score on the SCL-90 was 119.46. The general characteristics of the participants are presented in Table 1. There were no significant differences between participants with and without mental health issues in terms of gender, age, BMI, smoking status, alcohol consumption, diabetes, hypertension, and dyslipidemia. However, the education level was significantly higher in the group without mental health issues than in the group with mental health issues (p < 0.001). Additionally, the PLR and TyG index in the group without mental health issues were significantly lower than those in the group with mental health issues (p < 0.05).

**Table 1:** General information characteristics.

Variables	Overall(n=725)	Mental health problem		P
		Yes(n=150)	No(n=575)	
Age	52.54±11.56	51.71±11.92	52.75±11.46	0.246
Gender				0.380
Male	400(55.17)	78(52.00)	322(56.00)	
Female	325(44.83)	72(48.00)	253(44.00)	
BMI				0.298
Underweight	13(1.79)	1(0.67)	12(2.09)	
Normal weight	280(38.62)	55(36.67)	225(39.13)	
Overweight	278(38.34)	55(36.67)	223(38.78)	
Obesity	154(21.24)	39(26.00)	115(20.00)	
Smoke				0.415
Yes	74(10.21)	18(12.00)	56(9.74)	
No	651(89.79)	132(88.00)	519(90.26)	
Alcohol				0.969
Yes	149(20.55)	31(20.67)	118(20.52)	
No	576(79.45)	119(79.33)	457(79.48)	
Education level				<0.001
Junior high school or below	53(7.31)	19(12.67)	34(5.91)	
High school	73(10.07)	26(17.33)	47(8.17)	
College degree	280(38.62)	71(47.33)	209(36.35)	
College degree or above	319(44.00)	34(22.67)	285(49.57)	
Diabetes				1.000
Yes	58(8.00)	12(8.00)	46(8.00)	
No	667(92.00)	138(92.00)	529(92.00)	
Hypertension				0.318
Yes	272(37.52)	51(34.00)	221(338.43)	
No	453(62.48)	99(66.00)	354(61.57)	
Dyslipidemia				0.056
Yes	346(47.72)	82(54.67)	264(45.91)	
No	379(52.28)	68(45.33)	311(54.09)	
PLR	139.58±48.10	150.36±57.13	136.76±45.09	0.008
TyG Index	8.58±0.61	8.63±0.62	8.57±0.60	0.016
SCL-90 score	119.46±27.45	161.24±28.99	108.56±12.53	<0.001

Note: PLR, Platelet-lymphocyte ratio; TC, Total Cholesterol; LDL-C, Low-Density Lipoprotein Cholesterol; TG, Triglyceride; FBG, Fasting Blood Glucose; Triglyceride-Glucose Index

**Fig 1:**
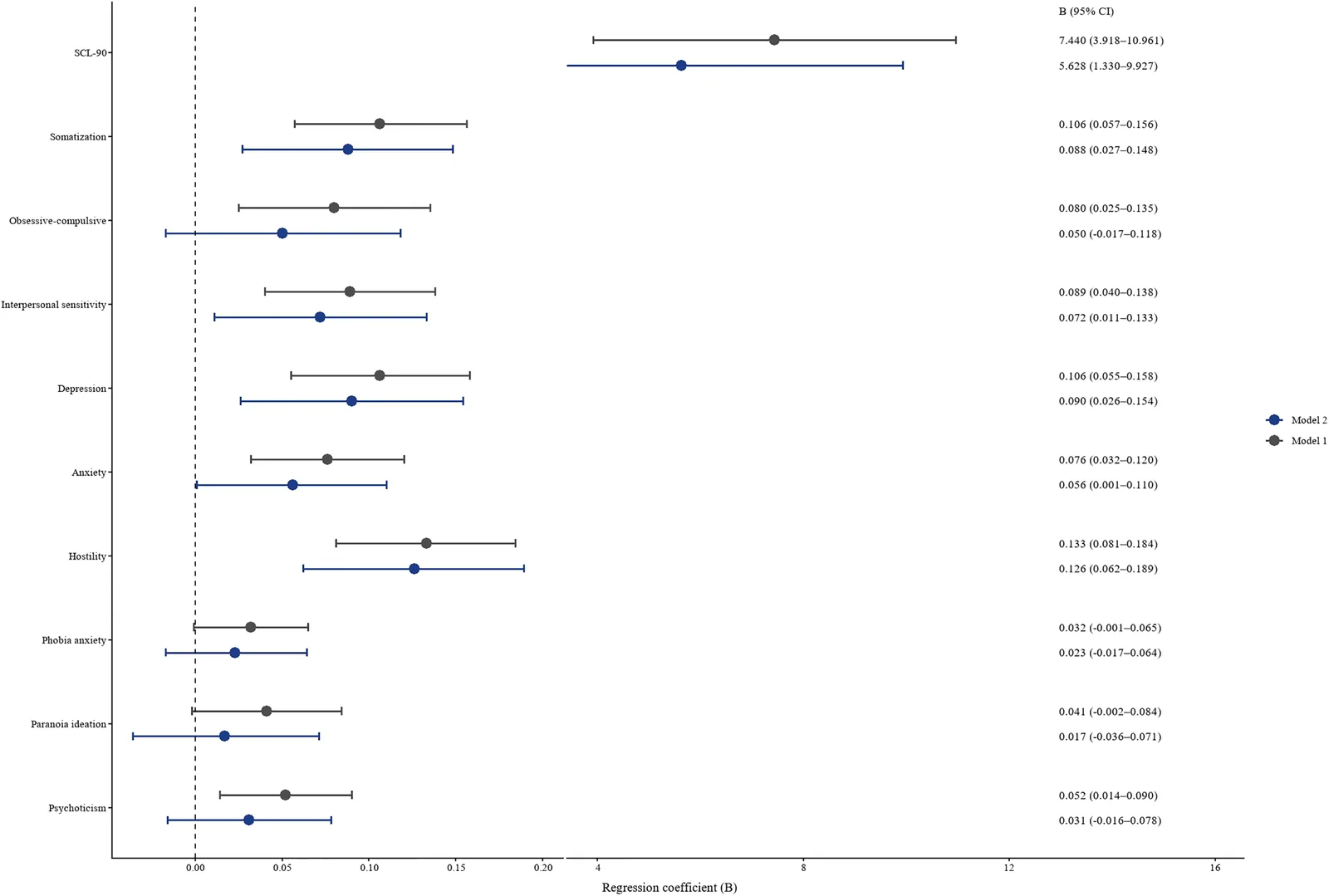
Association between TyG index and total score and sub-dimensions of SCL-90.

Fig 1 shows the independent associations of the TyG index with the total SCL-90 score and its sub-dimensions. Multiple linear regression analysis revealed that, in Model 2, the TyG index was significantly and positively associated with the total SCL-90 score, somatization, interpersonal sensitivity, depression, anxiety, and hostility (P < 0.05). However, no significant association was observed between the TyG index and obsessive-compulsive, phobic anxiety, paranoid ideation, or psychoticism (P > 0.05).

**Fig 2:**
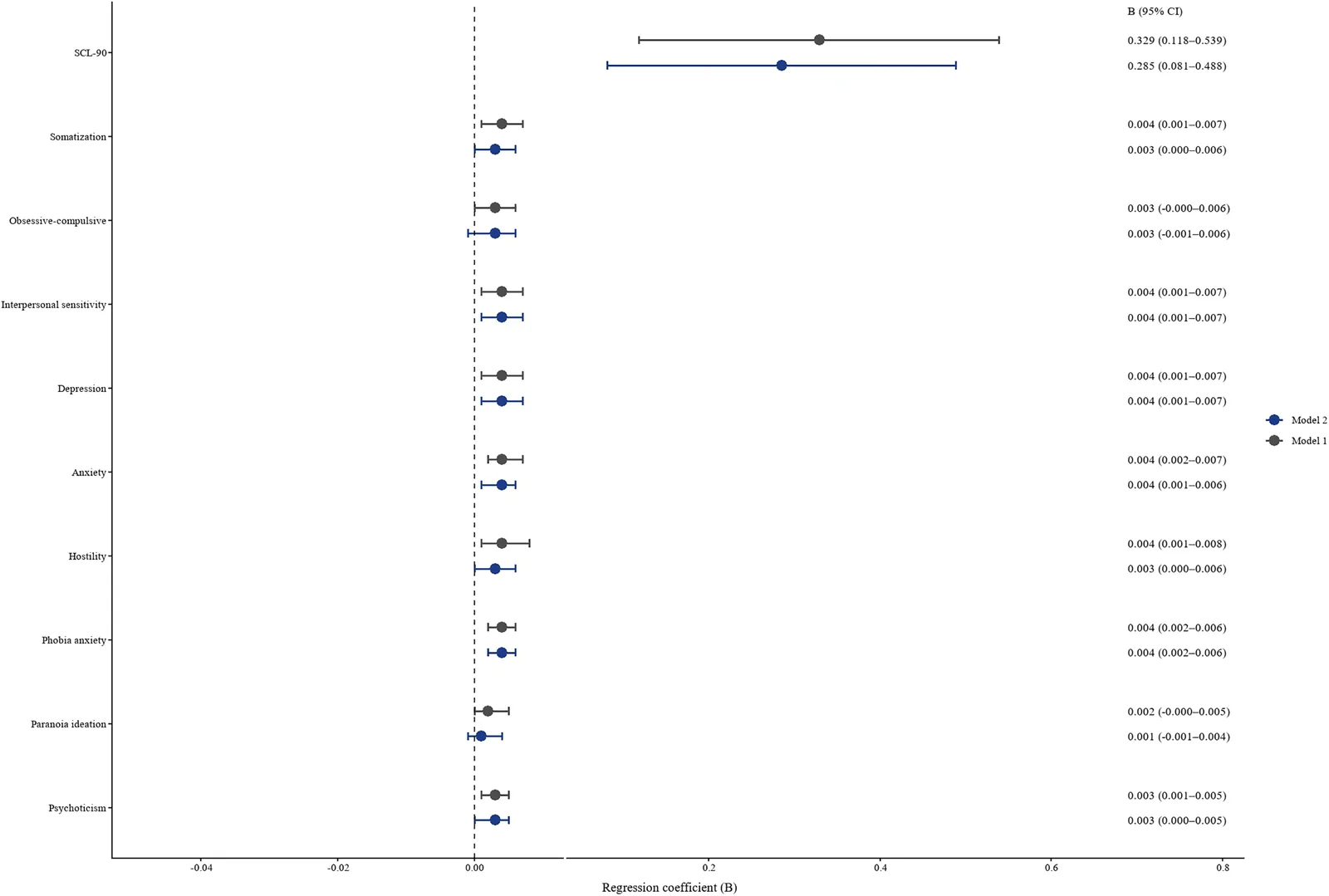
Association between TyG index and total score and sub-dimensions of SCL-90.

Fig 2 shows the independent associations of PLR with the total SCL-90 score and its sub-dimensions. Multiple linear regression analysis revealed that in Model 2, PLR was significantly and positively associated with the total SCL-90 score, as well as with somatization, interpersonal sensitivity, depression, anxiety, hostility, phobic anxiety, and psychoticism (P < 0.05). However, no significant association was observed between PLR and obsessive-compulsive or paranoid ideation (P > 0.05).

Table 2 shows the combined associations of different TyG index and PLR combinations with the total SCL-90 score and its sub-dimensions. Multiple linear regression analysis revealed that, compared with the low TyG/low PLR group, the high TyG/high PLR group exhibited significantly higher scores in the total SCL-90 score, somatization, interpersonal sensitivity, depression, anxiety, hostility, phobic anxiety, and psychoticism (P < 0.05). However, no significant association was observed between the high TyG/high PLR group and obsessive-compulsive or paranoid ideation (P > 0.05). Neither the low TyG/high PLR group nor the high TyG/low PLR group showed significant differences in the total SCL-90 score or its sub-dimensions (P > 0.05).

**Table 2:** Combined association of different combinations of TyG and PLR with the total score and sub-dimensions of SCL-90.

		Model 1		Model 2	
		B(95% CI)	P	B(95% CI)	P
SCL-90	Low TyG/low PLR	Ref	Ref	Ref	Ref
	Low TyG/high PLR	1.476(-4.169,7.120)	0.608	-0.460(-5.921,5.000)	0.869
	High TyG/low PLR	4.447(-1.362,10.257)	0.133	2.504(-3.559,8.568)	0.418
	High TyG/high PLR	13.350(7.323,19.376)	0.000	9.748(3.361,16.133)	0.003
Somatization	Low TyG/low PLR	Ref	Ref	Ref	Ref
	Low TyG/high PLR	0.022(-0.058,0.102)	0.588	-0.004(-0.081,0.073)	0.923
	High TyG/low PLR	0.068(-0.014,0.150)	0.104	0.046(-0.039,0.131)	0.289
	High TyG/high PLR	0.172(0.086,0.257)	0.000	0.126(0.036,0.215)	0.006
Obsessive-compulsive	Low TyG/low PLR	Ref	Ref	Ref	Ref
	Low TyG/high PLR	-0.027(-0.114,0.060)	0.545	-0.047(-0.133,0.038)	0.280
	High TyG/low PLR	0.012(-0.078,0.102)	0.789	-0.013(-0.108,0.082)	0.786
	High TyG/high PLR	0.144(0.051,0.238)	0.002	0.094(-0.006,0.194)	0.066
Interpersonal sensitivity	Low TyG/low PLR	Ref	Ref	Ref	Ref
	Low TyG/high PLR	0.030(-0.049,0.108)	0.456	0.010(-0.068,0.087)	0.805
	High TyG/low PLR	0.074(-0.007,0.155)	0.072	0.054(-0.032,0.140)	0.216
	High TyG/high PLR	0.155(0.071,0.239)	0.000	0.117(0.026,0.207)	0.011
Depression	Low TyG/low PLR	Ref	Ref	Ref	Ref
	Low TyG/high PLR	-0.007(-0.090,0.075)	0.861	-0.029(-0.111,0.052)	0.477
	High TyG/low PLR	0.056(-0.029,0.141)	0.193	0.040(-0.051,0.131)	0.388
	High TyG/high PLR	0.175(0.087,0.263)	0.000	0.142(0.047,0.238)	0.003
Anxiety	Low TyG/low PLR	Ref	Ref	Ref	Ref
	Low TyG/high PLR	0.053(-0.017,0.124)	0.136	0.035(-0.035,0.104)	0.331
	High TyG/low PLR	0.082(0.009,0.154)	0.027	0.059(-0.019,0.136)	0.136
	High TyG/high PLR	0.153(0.078,0.228)	0.000	0.117(0.036,0.199)	0.005
Hostility	Low TyG/low PLR	Ref	Ref	Ref	Ref
	Low TyG/high PLR	0.052(-0.031,0.136)	0.219	0.016(-0.065,0.097)	0.698
	High TyG/low PLR	0.099(0.013,0.185)	0.024	0.078(-0.012,0.168)	0.088
	High TyG/high PLR	0.215(0.126,0.305)	0.000	0.173(0.079,0.268)	0.000
Phobia anxiety	Low TyG/low PLR	Ref	Ref	Ref	Ref
	Low TyG/high PLR	0.032(-0.020,0.085)	0.226	0.020(-0.031,0.073)	0.441
	High TyG/low PLR	0.033(-0.021,0.086)	0.235	0.030(-0.028,0.088)	0.307
	High TyG/high PLR	0.099(0.043,0.155)	0.001	0.085(0.025,0.146)	0.006
Paranoia ideation	Low TyG/low PLR	Ref	Ref	Ref	Ref
	Low TyG/high PLR	-0.003(-0.072,0.065)	0.924	-0.021(-0.089,0.047)	0.539
	High TyG/low PLR	-0.004(-0.075,0.066)	0.902	-0.032(-0.108,0.043)	0.399
	High TyG/high PLR	0.072(-0.001,0.146)	0.053	0.030(-0.049,0.110)	0.456
Psychoticism	Low TyG/low PLR	Ref	Ref	Ref	Ref
	Low TyG/high PLR	0.022(-0.039,0.083)	0.484	0.003(-0.057,0.063)	0.927
	High TyG/low PLR	0.038(-0.025,0.101)	0.235	0.018(-0.049,0.085)	0.601
	High TyG/high PLR	0.107(0.042,0.172)	0.001	0.074(0.004,0.145)	0.038

Note: Model 1 adjusts age and gender; Model 2 adjusts age, gender, BMI, smoke, alcohol, education level, diabetes, hypertension, dyslipidemia.

**Fig 3:**
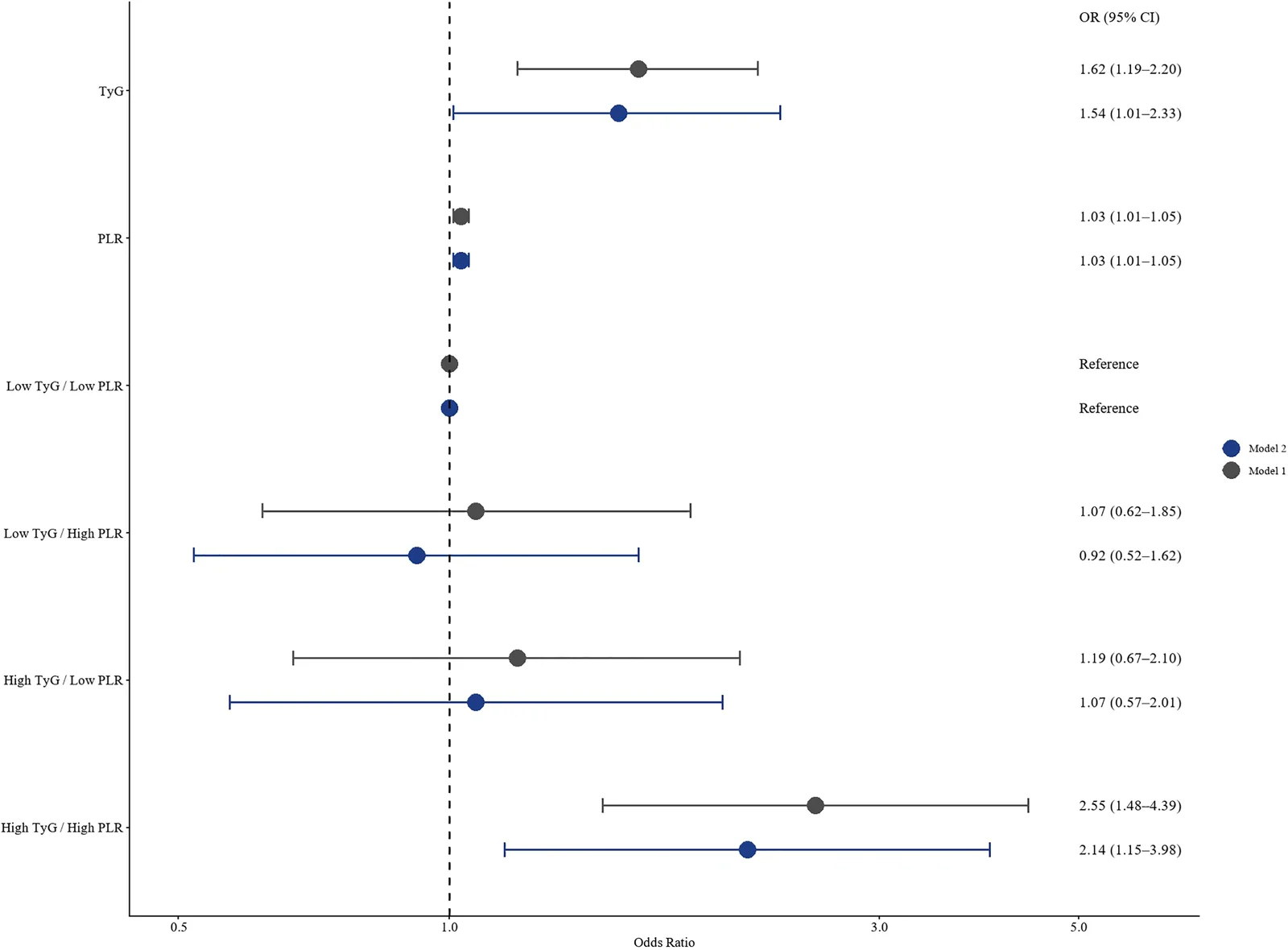
Independent and Combined associations of TyG and PLR with mental health problem.

Fig 3 presents the results of logistic regression analyses evaluating the independent and combined associations of the TyG index and PLR with mental health problem. In the analysis of independent associations (Model 2), both the TyG index (OR: 1.54, 95% CI: 1.01–2.33, P = 0.043) and PLR (OR: 1.03, 95% CI: 1.01–1.05, P = 0.008) were significantly associated with higher odds of poor mental health. Regarding combined associations, participants in the high TyG/high PLR group exhibited significantly higher odds of mental health problems compared to those in the low TyG/low PLR group (OR: 2.14, 95% CI: 1.15–3.98, P = 0.016). No statistically significant associations were observed for the low TyG/high PLR group (OR: 0.92, 95% CI: 0.52–1.62, P = 0.761) or the high TyG/low PLR group (OR: 1.07, 95% CI: 0.57–2.01, P = 0.839).

**Fig 4:**
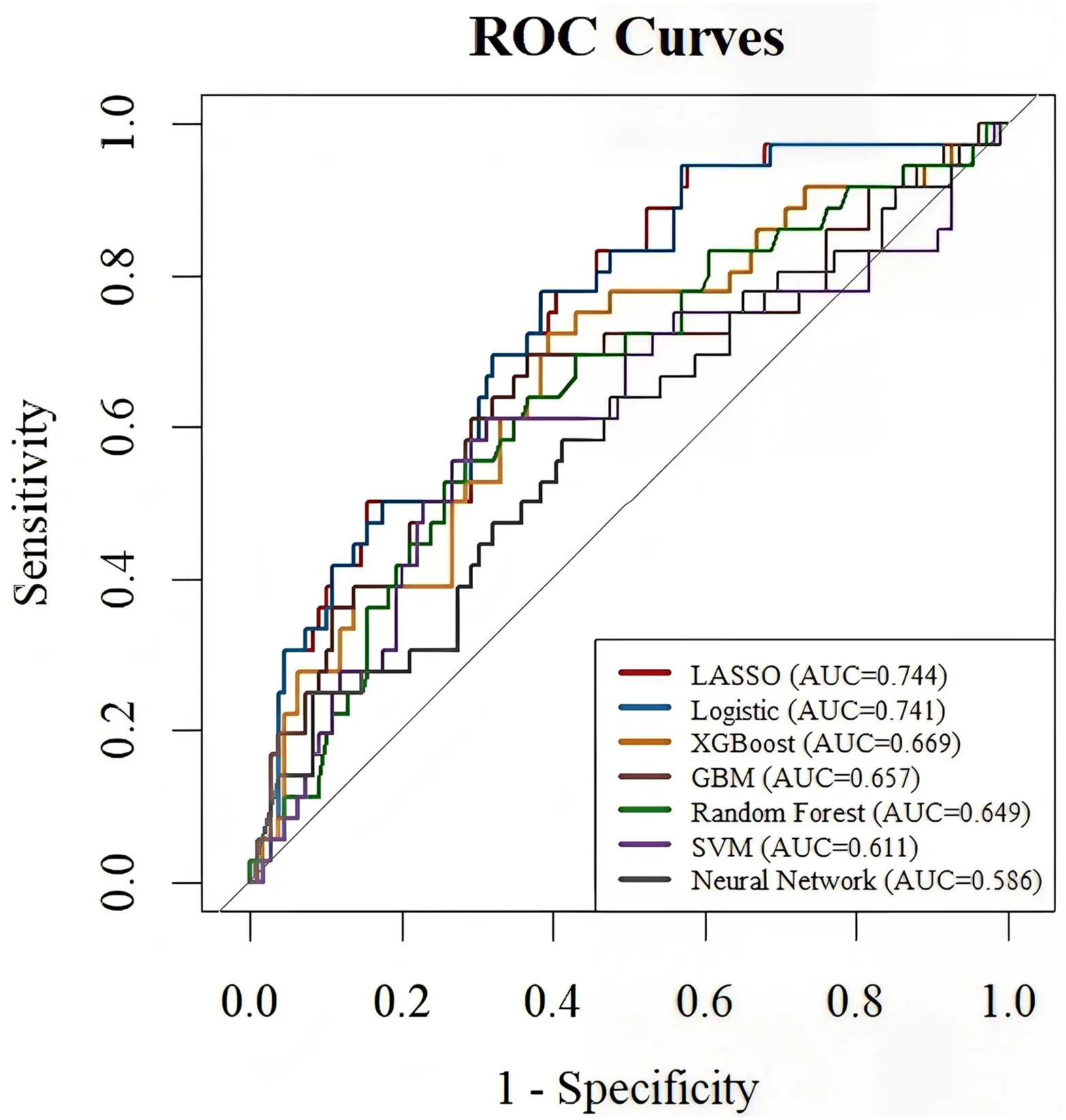
ROC curves of seven machine-learning models for mental health problems.

**Fig 5:**
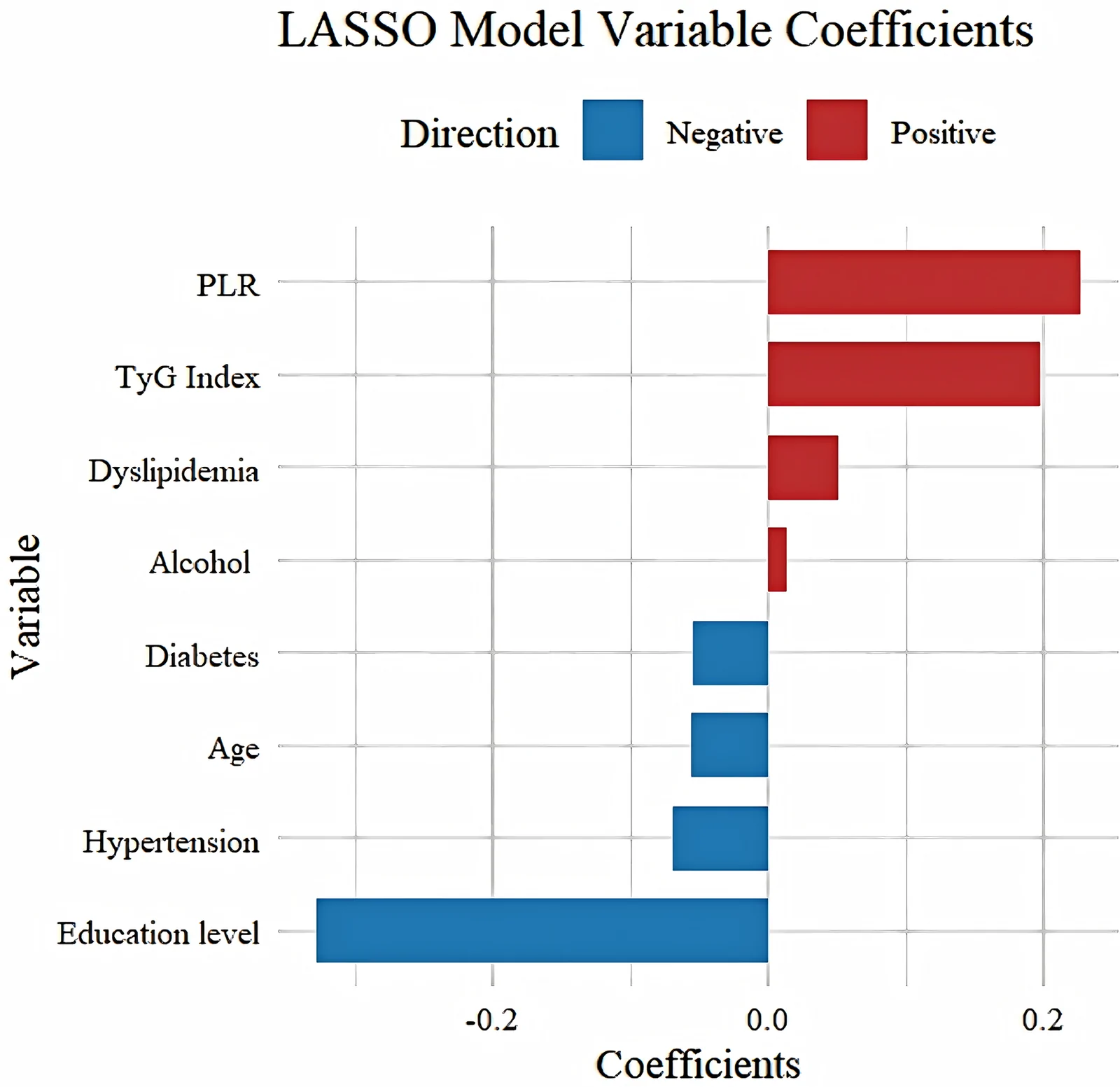
LASSO coefficient plot.

Seven machine-learning models were compared for predicting mental health problems (Fig 4, Table 3). LASSO regression achieved the best overall discrimination, with an AUC of 0.744, slightly higher than logistic regression (AUC = 0.741). XGBoost (AUC = 0.669), GBM (AUC = 0.657) and random forest (AUC = 0.649) showed moderate performance, while SVM (AUC = 0.611) and the neural network model (AUC = 0.586) performed less well. Across secondary metrics, LASSO showed the most balanced predictive performance, characterized by the highest F1-score (0.7826) and MCC (0.2500), along with favourable sensitivity (0.7431) and specificity (0.5278). In contrast, tree-based models exhibited higher sensitivity but substantially lower specificity. The LASSO coefficient plot (Fig 5) identified PLR and TyG index as the strongest positive predictors, whereas education level, hypertension, age and diabetes showed negative associations. Overall, LASSO regression demonstrated the best combination of discrimination, calibration, and model stability, and was therefore selected as the primary predictive model.

**Table 3:** Performance metrics of seven predictive models for mental health problem classification.

Models	Accuracy	Sensitivity	Specificity	F1	Brier Score	MCC	Kappa	Precision
LASSO Regression	0.6897	0.7431	0.5278	0.7826	0.2087	0.2500	0.2458	0.8265
Logistic regression	0.6897	0.7523	0.5000	0.7847	0.2057	0.2356	0.2328	0.8200
XGBoost	0.7379	0.8807	0.3056	0.8348	0.1893	0.2165	0.2097	0.7934
Random Forest	0.7034	0.8440	0.2778	0.8106	0.1919	0.1352	0.1329	0.7797
SVM	0.6828	0.8073	0.3056	0.7928	0.2115	0.1176	0.1173	0.7788
GBM	0.6966	0.7982	0.3889	0.7982	0.1959	0.1871	0.1871	0.7982
Neural Network	0.6345	0.6972	0.4444	0.7415	0.3513	0.1294	0.1264	0.7917

Note: Values represent accuracy, sensitivity, specificity, F1-score, Brier score, Matthews correlation coefficient (MCC), Cohen’s kappa, and precision derived from the validation dataset. Higher accuracy, sensitivity, F1-score, MCC, kappa, and precision indicate better predictive performance, whereas lower Brier scores indicate better calibration.

## 4. Discussion

This study examined the independent and combined associations of the TyG index and PLR with mental health in adults and assessed their predictive value using multiple machine learning models. The TyG index was associated with several key mental symptom domains, while PLR showed broader associations. Compared with the low-TyG/low-PLR group, the high-TyG/high-PLR group displayed higher SCL-90 total and subdimension scores. Among the prediction models, LASSO achieved the best performance (AUC = 0.744) with a balanced sensitivity, specificity, and F1-score. TyG index and PLR were consistently identified as the strongest predictors. Overall, the findings suggest that the TyG index and PLR may serve as useful indicators for characterizing mental health problem within a multidimensional predictive framework.

The TyG index, a simple biomarker derived from routine glucose and lipid measurements, has been widely validated as a reliable surrogate indicator of IR and is broadly used in metabolic research. In the present study, higher TyG levels were significantly associated with poorer overall mental health, as reflected by higher total SCL-90 scores and elevated scores in the depression and anxiety dimensions. These findings are consistent with prior evidence showing positive associations between HOMA-IR and depression or anxiety among adults in Korea and Ireland [34,35]. Studies using the TyG index as an IR surrogate have similarly reported significant positive associations between TyG and depressive and anxiety symptoms [14,15,36]. A recent meta-analysis further indicated that higher TyG levels are associated with an increased prevalence of depression in adults [37], providing additional external support for our results. In contrast to the consistent findings in adults, an 11-year follow-up study among older adults reported no association between TyG and subsequent depression [38]. This discrepancy suggests that the impact of insulin resistance on psychological health may be age-specific. The pathophysiology of late-life depression is highly complex and may be more strongly influenced by cognitive decline, age-related alterations in inflammatory pathways, shifts in metabolic phenotypes, and the cumulative burden of chronic diseases. Such heterogeneity may reduce the predictive capacity of a single metabolic marker such as TyG index in older populations. Notably, this study is the first to demonstrate that the TyG index is associated not only with depression and anxiety but also with somatization, interpersonal sensitivity, and hostility among Chinese adults. These findings have biological plausibility: as a surrogate for IR, elevated TyG reflects metabolic dysfunction that can promote chronic low-grade inflammation and oxidative stress [39], which may disrupt neurotransmitter metabolism and impair neuroplasticity, thereby contributing to depressive and anxiety symptoms [40]. In addition, IR can dysregulate the hypothalamic-pituitary-adrenal (HPA) axis, leading to abnormal cortisol secretion patterns that are closely associated with mood disorders and stress-related psychopathology [41,42]. Taken together, the TyG index may serve not only as a marker of metabolic dysfunction but also as a valuable tool for identifying mental health problem.

As an inflammatory marker derived directly from routine complete blood counts, PLR offers the distinct advantages of low cost and operational simplicity. In this study, PLR was significantly associated with overall mental health, particularly with the anxiety and depression dimensions—findings that are highly consistent with previous evidence. For example, a retrospective study reported that patients with major depressive disorder accompanied by psychotic features exhibited significantly higher PLR levels compared with those presenting non-psychotic depression [43]. Among adults with vitamin D deficiency, elevated PLR was positively associated with anxiety and depressive symptoms [44]. Similarly, in participants with COVID-19 infection, PLR was positively associated with depression and anxiety scores [25]. Furthermore, meta-analytic evidence and studies conducted in U.S. adults provide additional support for the positive association between PLR and depressive symptoms [45–47]. However, not all studies have observed significant associations. A randomized controlled trial in adolescents reported no association between PLR and depressive symptoms [48]; additionally, a prospective cohort study among full-term pregnant women also found no significant association between PLR and depression [49]. Likewise, a cross-sectional case-control study reported that PLR was not significantly associated with anxiety or depression severity in patients with major depressive disorder [50]. Such inconsistencies may arise from differences in sample characteristics (e.g., age, pregnancy-related hormonal changes, and disease severity) or insufficient sensitivity of mental assessment tools. Variations in study design are also important contributors: small-sample studies may be underpowered to detect small effect sizes, and randomized trials or studies targeting special populations may introduce selection bias or group imbalances. These findings suggest that the association between PLR and mental health may be population-dependent rather than universally generalizable. Furthermore, the present study identified significant associations between PLR and multiple mental symptom dimensions, including somatization, interpersonal sensitivity, hostility, phobic anxiety, and psychoticism—indicating that chronic inflammation may exert broad influences on psychological functioning beyond anxiety and depression alone. According to the inflammatory hypothesis of depression, proinflammatory cytokines (e.g., IL-6, TNF-α) can affect the prefrontal cortex, hippocampus, and amygdala through mechanisms such as crossing the blood-brain barrier or activating neuroimmune pathways, thereby altering emotion regulation, impulse control, and socioemotional processing [51]. Inflammation-induced disruptions in neurotransmitter metabolism (e.g., serotonin, dopamine) may contribute to cognitive disturbances and heightened stress or fear-related responses [52]. Chronic inflammation can also dysregulate the HPA axis, keeping the body in a prolonged stress response and thereby intensifying multidimensional psychological symptoms [53]. Taken together, our findings support the positive association between PLR and multiple psychological dimensions in adults, underscoring the potential role of inflammatory status in diverse psychological manifestations. These results further suggest that PLR may serve as a useful biomarker for multidimensional psychological symptoms and provide a theoretical basis for future anti-inflammatory or lifestyle-based interventions.

IR and systemic inflammation are intricately interconnected, often co-occurring at elevated levels [54]. Although previous studies have independently confirmed associations of IR or inflammation with various health outcomes, the combined effect of TyG index and PLR on mental health has not been systematically examined. In the present study, participants in the high TyG/high PLR group exhibited a significantly higher prevalence of mental health problems compared with the low TyG/low PLR group, with positive associations observed for total SCL-90 scores and most symptom dimensions. These findings suggest a potential synergistic effect of IR and inflammation, whereby their combined impact on psychological symptoms may exceed that of either marker alone. Notably, existing evidence supporting the “high IR + high inflammation” synergistic risk primarily comes from research on somatic diseases, such as cancer. For example, multicenter prospective studies have shown that cancer patients with concurrent high IR and high inflammatory levels experience worse prognoses [55]. A cohort study in Tangshan reported a 75% increase in cancer mortality risk among participants with high IR/high inflammation status [54], and similar synergistic effects have been observed in prospective studies of breast cancer [56]. Although these findings demonstrate cross-disease consistency, caution is warranted when extrapolating to mental health, as the underlying pathophysiology differs between mental and somatic disorders. Mechanistically, IR can induce glucose and lipid metabolic dysregulation and oxidative stress, which in turn can trigger systemic inflammation [27]. Inflammatory cytokines, such as IL-6 and TNF-α, can disrupt neurotransmitter metabolism, impair neuroplasticity, and dysregulate HPA axis function [57], potentially exacerbating mood and cognitive symptoms. The increased mental health odds observed in the high TyG/high PLR group is consistent with these biological mechanisms. In summary, this study is the first to demonstrate that the concurrent elevation of TyG and PLR is significantly associated with multidimensional psychological symptoms, providing novel evidence for the potential synergistic role of insulin resistance and chronic inflammation in mental health. Nevertheless, these findings require further validation in longitudinal studies with additional biomarkers and multicenter cohorts to strengthen causal inference, minimize overinterpretation, and provide a more robust foundation for early identification and intervention strategies.

In this study, the LASSO regression model was ultimately selected to predict poor mental health, as it offered the optimal balance among performance, interpretability, and clinical utility. Among the models compared, LASSO achieved the highest AUC in the test set while maintaining the best balance between sensitivity and specificity. Although XGBoost demonstrated higher sensitivity during training, its markedly lower specificity, together with the characteristics of its learning curves, suggests a heightened risk of overfitting in the context of a limited sample size, which may compromise external validity. The neural network model showed relatively modest performance, likely due to insufficient sample size and the lack of strong nonlinear interactions among predictors. Therefore, by prioritizing robustness and clinical interpretability, LASSO—with its L1 regularization, automatic feature selection, and transparent linear coefficient structure—proved to be the most suitable modeling approach for this study. The LASSO coefficient results identified the TyG index and PLR as the most critical predictors of poor psychological health. This finding is consistent with previous evidence supporting the predictive value of TyG and PLR in somatic disease contexts such as diabetic complications [58,59] and extends their application to the domain of mental health problem prediction. Additionally, socioeconomic and cardiometabolic variables, including educational attainment, hypertension, and diabetes also made meaningful contributions to prediction, underscoring the multifactorial nature of psychological health. It is important to note, however, that LASSO, as a linear modeling approach, is limited in its ability to capture nonlinear relationships or higher-order interactions among predictors. Although LASSO achieved the highest AUC and the most favorable balance between sensitivity and specificity in our sample, this performance likely reflects the modest sample size, which constrains the generalizability of more complex algorithms and increases the risk of overfitting. Therefore, despite the substantial linear contributions of TyG and PLR observed in our findings, potential nonlinear patterns and interactions may still exist and should be examined in larger, multicenter cohorts. Importantly, the interpretability of LASSO offers a practical advantage in clinical settings. Its linear coefficients enable direct quantification of the contributions of TyG, PLR, and other covariates, thereby facilitating actionable and transparent risk stratification. Compared with more opaque “black-box” models, this transparency promotes clinical understanding and implementation, ensuring that predictive insights can be effectively translated into early identification and intervention strategies within primary care or resource-limited settings.

The findings of this study suggest that the TyG index and PLR, as routinely obtainable biomarkers, hold meaningful potential for identifying participants at elevated odds for mental health problems. In clinical practice, these indicators can serve as objective signals for primary care physicians, helping them identify individuals, especially those showing concurrent abnormalities in both TyG and PLR, who may require more comprehensive psychological evaluation using standardized depression and anxiety scales. This approach can facilitate earlier detection and timely management of mental health concerns. From a public health perspective, incorporating these biomarkers into population-level health surveillance systems may aid in the preliminary stratification of psychological risk from metabolic and inflammatory dimensions, thereby providing valuable information for targeted resource allocation and planning. However, the applicability of these findings to other populations should be interpreted with caution. Although insulin resistance and chronic inflammation have been broadly supported across cultures as potential neuropsychiatric pathophysiological mechanisms, the magnitude, consistency, and manifestation of associations between specific biomarkers (such as TyG and PLR) and psychological symptom phenotypes may be shaped by sociocultural contexts, lifestyle patterns, and healthcare system differences. For instance, the significant association with somatization observed in this study may reflect a culturally characteristic mode of expressing psychological distress within the Chinese context; in other cultural settings, similar biological vulnerabilities may present with distinct symptom patterns. Therefore, caution is warranted when extrapolating the potential applications of this study to other populations.

The strength of this study lies in its extension of the associations of TyG and PLR with mental health to multi-dimensional psychological symptoms such as somatization and interpersonal sensitivity, which are commonly observed in the Chinese population. Undoubtedly, this study has several limitations that should be acknowledged. First, the cross-sectional design limits our ability to infer causal relationships among the variables examined; therefore, observed associations should be interpreted as correlational rather than causal. Second, although the study included 725 participants, the sample size remains moderate and may limit statistical power to detect more subtle associations. Third, the single-center sampling framework may reduce the representativeness of the study population, thereby constraining the generalizability of the findings to broader or more diverse populations. Fourth, despite adjustment for key covariates, some unmeasured or residual confounding may still exist and could influence the observed associations. Fifth, although cross-validation was conducted, multi-model comparisons may still introduce a risk of overfitting, and the interpretability of some machine learning methods remains limited. Future research should employ multi-center cohorts with larger and more heterogeneous samples and adopt longitudinal designs to enhance external validity, improve causal inference, and further verify the robustness of the findings.

## 5. Conclusion

Both the TyG index and PLR were positively associated with mental health problem among Chinese adults, showing consistent patterns across multiple SCL-90 subdimensions. In the combined analyses, participants with both high TyG/high PLR exhibited higher psychological symptom scores and increased odds of mental health problems. In the LASSO model, the TyG index and PLR demonstrated favorable diagnostic performance in predicting mental health problem, suggesting that these biomarkers may serve as useful indicators for identifying individuals at heightened psychological health risk.

## Supporting information

S1 Data Dataset underlying the findings of this study. The Excel file contains the de-identified participant-level data used in the statistical and machine learning analyses.
(XLSX)

S1 File The Word file contains Table S1, which summarizes the dataset variables, variable types, coding, and roles in the analysis, and Table S2, which presents the parameters of the machine learning models. (DOCX)

## References

[1] Allsop AS, Tye KM, Krystal JH. Social Homeostasis: A New Paradigm for Mental Health Diagnosis and Treatment. Biol Psychiatry. 2025;97(10):932–5. doi: 10.1016/j.biopsych.2025.03.007 40316377

[2] Patel V, Saxena S, Lund C, Kohrt B, Kieling C, Sunkel C, et al. Transforming mental health systems globally: principles and policy recommendations. Lancet. 2023;402(10402):656–66. doi: 10.1016/S0140-6736(23)00918-2 37597892

[3] Shan Y, Liu L, Wang F, Yu L, Liu D, Zheng C, et al. Association between inequalities in mental health resources and burdens of mental health disorders in 146 countries and territories: An observational study. J Affect Disord. 2025;379:812–21. doi: 10.1016/j.jad.2025.03.094 40113180

[4] Herrman H, Patel V, Kieling C, Berk M, Buchweitz C, Cuijpers P, et al. Time for united action on depression: a Lancet-World Psychiatric Association Commission. Lancet. 2022;399(10328):957–1022. doi: 10.1016/S0140-6736(21)02141-3 35180424

[5] Arias D, Saxena S, Verguet S. Quantifying the global burden of mental disorders and their economic value. EClinicalMedicine. 2022;54:101675. doi: 10.1016/j.eclinm.2022.101675 36193171 PMC9526145

[6] Lebovitz HE. Insulin resistance: definition and consequences. Exp Clin Endocrinol Diabetes. 2001;109 Suppl 2:S135-48. doi: 10.1055/s-2001-18576 11460565

[7] Moon S, Choi JW, Park JH, Kim DS, Ahn Y, Kim Y, et al. Association of Appendicular Skeletal Muscle Mass Index and Insulin Resistance With Mortality in Multi-Nationwide Cohorts. J Cachexia Sarcopenia Muscle. 2025;16(2):e13811. doi: 10.1002/jcsm.13811 40230053 PMC11997253

[8] Yang D, Zhou J, Garstka MA, Xu Q, Li Q, Wang L, et al. Association of obesity- and insulin resistance-related indices with subclinical carotid atherosclerosis in type 1 diabetes: a cross-sectional study. Cardiovasc Diabetol. 2025;24(1):193. doi: 10.1186/s12933-025-02736-2 40319311 PMC12049799

[9] Tao S, Yu L, Li J, Wu J, Huang X, Xie Z, et al. Insulin resistance quantified by estimated glucose disposal rate predicts cardiovascular disease incidence: a nationwide prospective cohort study. Cardiovasc Diabetol. 2025;24(1):161. doi: 10.1186/s12933-025-02672-1 40223076 PMC11995552

[10] Ntetsika T, Catrina S-B, Markaki I. Understanding the link between type 2 diabetes mellitus and Parkinson’s disease: role of brain insulin resistance. Neural Regen Res. 2025;20(11):3113–23. doi: 10.4103/NRR.NRR-D-23-01910 39715083 PMC11881720

[11] Hanssen R, Bouzouina A, Reif A, Edwin Thanarajah S. Connecting the dots: Insulin resistance and mental health. Neurosci Biobehav Rev. 2024;158:105549. doi: 10.1016/j.neubiorev.2024.105549 38242521

[12] DeFronzo RA, Tobin JD, Andres R. Glucose clamp technique: a method for quantifying insulin secretion and resistance. Am J Physiol. 1979;237(3):E214-23. doi: 10.1152/ajpendo.1979.237.3.E214 382871

[13] Zuo Z, Zhou Z, Liu Q, Shi R, Wu T. Joint association of the triglyceride-glucose index and stress hyperglycemia ratio with incidence and mortality risks of new-onset atrial fibrillation during sepsis: a retrospective cohort study. Cardiovasc Diabetol. 2025;24(1):149. doi: 10.1186/s12933-025-02709-5 40176089 PMC11966863

[14] Wang Y, Wang H, Cheng B, Xia J. Associations between triglyceride glucose index-related obesity indices and anxiety: Insights from the National Health and Nutrition Examination Survey 2007-2012. J Affect Disord. 2025;382:443–52. doi: 10.1016/j.jad.2025.04.134 40280441

[15] Ding C, Lu R, Kong Z, Huang R. Exploring the triglyceride-glucose index’s role in depression and cognitive dysfunction: Evidence from NHANES with machine learning support. J Affect Disord. 2025;374:282–9. doi: 10.1016/j.jad.2025.01.051 39805501

[16] Zaroff CM, Davis JM, Chio PH, Madhavan D. Somatic presentations of distress in China. Aust N Z J Psychiatry. 2012;46(11):1053–7. doi: 10.1177/0004867412450077 22696549

[17] Choi E, Chentsova-Dutton Y, Parrott WG. The Effectiveness of Somatization in Communicating Distress in Korean and American Cultural Contexts. Front Psychol. 2016;7:383. doi: 10.3389/fpsyg.2016.00383 27047414 PMC4803738

[18] Lee STH. Inflammation, depression, and anxiety disorder: A population-based study examining the association between Interleukin-6 and the experiencing of depressive and anxiety symptoms. Psychiatry Res. 2020;285:112809. doi: 10.1016/j.psychres.2020.112809 32045822

[19] Eisenberger NI, Moieni M. Inflammation affects social experience: implications for mental health. World Psychiatry: Official Journal of the World Psychiatric Association (Wpa). 2020;19(1):109–10. doi: 10.1002/wps.20724 PMC6953558 31922673

[20] Hao R, Gao X, Lu Q, Zhao T, Lu X, Zhang F, et al. CUMS induces depressive-like behaviors and cognition impairment by activating the ERS-NLRP3 signaling pathway in mice. J Affect Disord. 2025;369:547–58. doi: 10.1016/j.jad.2024.10.001 39378914

[21] Van Assche E, Hohoff C, Su Atil E, Wissing SM, Serretti A, Fabbri C, et al. Exploring the use of immunomethylomics in the characterization of depressed patients: A proof-of-concept study. Brain Behav Immun. 2025;123:597–605. doi: 10.1016/j.bbi.2024.09.026 39341467

[22] Valkanova V, Ebmeier KP, Allan CL. CRP, IL-6 and depression: a systematic review and meta-analysis of longitudinal studies. J Affect Disord. 2013;150(3):736–44. doi: 10.1016/j.jad.2013.06.004 23870425

[23] Gasparyan AY, Ayvazyan L, Mukanova U, Yessirkepov M, Kitas GD. The Platelet-to-Lymphocyte Ratio as an Inflammatory Marker in Rheumatic Diseases. Ann Lab Med. 2019;39(4):345–57. doi: 10.3343/alm.2019.39.4.345 30809980 PMC6400713

[24] Lee YH, Song GG. Platelet-to-lymphocyte ratio as a biomarker of systemic inflammation in systemic lupus erythematosus: A meta-analysis and systematic review. PLoS One. 2024;19(5):e0303665. doi: 10.1371/journal.pone.0303665 38753735 PMC11098385

[25] Khorasanchi Z, Rashidmayvan M, Hasanzadeh E, Moghadam MRSF, Afkhami N, Asadiyan-Sohan P, et al. The association of hematological inflammatory markers and psychological function in COVID-19 patients: A cross-sectional study. Physiol Rep. 2023;11(24):e15889. doi: 10.14814/phy2.15889 38123447 PMC10733126

[26] Li H, Meng Y, He S, Tan X, Zhang Y, Zhang X. Macrophages, chronic inflammation, and insulin resistance. Cells. 2022;11(19). doi: 10.3390/cells11193001 PMC9562180 36230963

[27] Chen L, Chen R, Wang H, Liang F. Mechanisms Linking Inflammation to Insulin Resistance. Int J Endocrinol. 2015;2015:508409. doi: 10.1155/2015/508409 26136779 PMC4468292

[28] Ader R, Cohen N, Felten D. Psychoneuroimmunology: interactions between the nervous system and the immune system. Lancet. 1995;345(8942):99–103. 7815892 10.1016/s0140-6736(95)90066-7

[29] McEwen BS. Stress and the Individual. Arch Intern Med. 1993;153(18):2093. doi: 10.1001/archinte.1993.00410180039004 8379800

[30] Dantzer R, O’Connor JC, Freund GG, Johnson RW, Kelley KW. From inflammation to sickness and depression: when the immune system subjugates the brain. Nat Rev Neurosci. 2008;9(1):46–56. doi: 10.1038/nrn2297 18073775 PMC2919277

[31] Han M, Shi X, Xiong D, Zhang X, Shen X, Wu N, et al. The status and influencing factors of adolescents’ mental health in a province of China: A cross-sectional study. J Affect Disord. 2023;321:41–6. doi: 10.1016/j.jad.2022.10.024 36273679

[32] Turkmen K, Erdur FM, Ozcicek F, Ozcicek A, Akbas EM, Ozbicer A, et al. Platelet-to-lymphocyte ratio better predicts inflammation than neutrophil-to-lymphocyte ratio in end-stage renal disease patients. Hemodial Int. 2013;17(3):391–6. doi: 10.1111/hdi.12040 23522328

[33] Yaprak M, Turan MN, Dayanan R, Akın S, Değirmen E, Yıldırım M, et al. Platelet-to-lymphocyte ratio predicts mortality better than neutrophil-to-lymphocyte ratio in hemodialysis patients. Int Urol Nephrol. 2016;48(8):1343–8. doi: 10.1007/s11255-016-1301-4 27118565

[34] Lee J-H, Park SK, Ryoo J-H, Oh C-M, Mansur RB, Alfonsi JE, et al. The association between insulin resistance and depression in the Korean general population. J Affect Disord. 2017;208:553–9. doi: 10.1016/j.jad.2016.10.027 27810270

[35] Phillips CM, Perry IJ. Depressive symptoms, anxiety and well-being among metabolic health obese subtypes. Psychoneuroendocrinology. 2015;62:47–53. doi: 10.1016/j.psyneuen.2015.07.168 26232649

[36] Liu P-L, Gao S, Zhang Y, Li J, Du J, Dong Q-L, et al. Associations between the triglyceride-glucose index and its combined indices with sleep disorders and the mediating role of depressive symptoms: Results from two nationally representative population studies. J Affect Disord. 2025;386:119480. doi: 10.1016/j.jad.2025.119480 40419145

[37] Wan W, Yu Y. Association between the triglyceride glucose index and depression: a meta-analysis. Front Psychiatry. 2024;15:1390631. doi: 10.3389/fpsyt.2024.1390631 38966187 PMC11222386

[38] Forbes M, Mohebbi M, Woods RL, Lotfaliany M, Reynolds CF 3rd, O’Neil A, et al. Triglyceride-glucose index and its association with depressive symptoms in older adults: a longitudinal analysis. J Affect Disord. 2025;384:80–5. doi: 10.1016/j.jad.2025.05.014 40334858

[39] Leonard BE, Wegener G. Inflammation, insulin resistance and neuroprogression in depression. Acta Neuropsychiatr. 2020;32(1):1–9. doi: 10.1017/neu.2019.17 31186075

[40] Beurel E, Toups M, Nemeroff CB. The Bidirectional Relationship of Depression and Inflammation: Double Trouble. Neuron. 2020;107(2):234–56. doi: 10.1016/j.neuron.2020.06.002 32553197 PMC7381373

[41] Joseph JJ, Golden SH. Cortisol dysregulation: the bidirectional link between stress, depression, and type 2 diabetes mellitus. Ann N Y Acad Sci. 2017;1391(1):20–34. doi: 10.1111/nyas.13217 27750377 PMC5334212

[42] Hantsoo L, Jagodnik KM, Novick AM, Baweja R, di Scalea TL, Ozerdem A, et al. The role of the hypothalamic-pituitary-adrenal axis in depression across the female reproductive lifecycle: current knowledge and future directions. Front Endocrinol (Lausanne). 2023;14:1295261. doi: 10.3389/fendo.2023.1295261 38149098 PMC10750128

[43] Kayhan F, Gündüz Ş, Ersoy SA, Kandeğer A, Annagür BB. Relationships of neutrophil-lymphocyte and platelet-lymphocyte ratios with the severity of major depression. Psychiatry Res. 2017;247:332–5. doi: 10.1016/j.psychres.2016.11.016 27978453

[44] Sharifan P, Darroudi S, Rafiee M, Toussi MSE, Sedgh Doust FN, Taghizadeh N, et al. Association of dietary and blood inflammatory indicators with depression, anxiety, and stress in adults with vitamin D deficiency. Int J Geriatr Psychiatry. 2023;38(8):e5972. doi: 10.1002/gps.5972 37539817

[45] Cheng Y, Wang Y, Wang X, Jiang Z, Zhu L, Fang S. Neutrophil-to-Lymphocyte Ratio, Platelet-to-Lymphocyte Ratio, and Monocyte-to-Lymphocyte Ratio in Depression: An Updated Systematic Review and Meta-Analysis. Front Psychiatry. 2022;13:893097. doi: 10.3389/fpsyt.2022.893097 35782448 PMC9240476

[46] Zhu M, Li W. Association of platelet-to-lymphocyte ratio with depression risk: a systematic review and meta-analysis. Front Psychiatry. 2025;16:1671777. doi: 10.3389/fpsyt.2025.1671777 41200231 PMC12586146

[47] Shan M, Yang Z, Sun Z, Yang Y, Cheng Q, Pan Y. Association between platelet to lymphocyte ratio and depression and symptom severity among adults in the United States: A cross-sectional study. Heliyon. 2023;9(9):e20127. doi: 10.1016/j.heliyon.2023.e20127 37809517 PMC10559847

[48] Ucar HN, Eray S, Murat D. Simple peripheral markers for inflammation in adolescents with major depressive disorder. Psychiat Clin Psych. 2018;28(3):254–60. doi: 10.1080/24750573.2018.1423769

[49] La Verde M, Luciano M, Fordellone M, Sampogna G, Lettieri D, Palma M, et al. Postpartum Depression and Inflammatory Biomarkers of Neutrophil-Lymphocyte Ratio, Platelet-Lymphocyte Ratio, and Monocyte-Lymphocyte Ratio: A Prospective Observational Study. Gynecol Obstet Inves. 2024;89(2):140–9. doi: 10.1159/000536559 38346412

[50] Liu Y, Li C, Ren H, Han K, Wang X, Zang S, et al. The relationship of peripheral blood cell inflammatory biomarkers and psychological stress in unmedicated major depressive disorder. J Psychiatr Res. 2024;176:155–62. doi: 10.1016/j.jpsychires.2024.06.013 38865865

[51] Liu B, Yuan M-L, Hu Y, Ge F-F, Wang J-Y, Zhang W. A Review of Research Progress in the Pathophysiological Mechanism of Stress-related Mental Disorders. Sichuan Da Xue Xue Bao Yi Xue Ban. 2021;52(1):22–7. doi: 10.12182/20210160101 33474884 PMC10408935

[52] Miller AH, Raison CL. The role of inflammation in depression: from evolutionary imperative to modern treatment target. Nat Rev Immunol. 2016;16(1):22–34. doi: 10.1038/nri.2015.5 26711676 PMC5542678

[53] Smith SM, Vale WW. The role of the hypothalamic-pituitary-adrenal axis in neuroendocrine responses to stress. Dialogues Clin Neurosci. 2006;8(4):383–95. doi: 10.31887/DCNS.2006.8.4/ssmith 17290797 PMC3181830

[54] Zheng X, Wang Y, Chen Y, Liu C, Tong L, Lin S, et al. Temporal relationship between chronic inflammation and insulin resistance and their combined cumulative effect on cancer risk: a longitudinal cohort study. BMC Public Health. 2025;25(1):1501. doi: 10.1186/s12889-025-22632-4 40269845 PMC12016061

[55] Ruan G-T, Xie H-L, Gong Y-Z, Ge Y-Z, Zhang Q, Wang Z-W, et al. Prognostic importance of systemic inflammation and insulin resistance in patients with cancer: a prospective multicenter study. BMC Cancer. 2022;22(1):700. doi: 10.1186/s12885-022-09752-5 35752767 PMC9233357

[56] Ruan G-T, Xie H-L, Hu C-L, Liu C-A, Zhang H-Y, Zhang Q, et al. Comprehensive prognostic effects of systemic inflammation and Insulin resistance in women with breast cancer with different BMI: a prospective multicenter cohort. Sci Rep. 2023;13(1):4303. doi: 10.1038/s41598-023-31450-w 36922570 PMC10017691

[57] Mehdi S, Wani SUD, Krishna KL, Kinattingal N, Roohi TF. A review on linking stress, depression, and insulin resistance via low-grade chronic inflammation. Biochem Biophys Rep. 2023;36:101571. doi: 10.1016/j.bbrep.2023.101571 37965066 PMC10641573

[58] Li J, Li Y, Qi Q, Chen N, Zhang Y. Predictive value of triglyceride glucose index and systemic inflammation index for diabetic retinopathy in Type-2 diabetes. Pak J Med Sci. 2025;41(4):1072–7. doi: 10.12669/pjms.41.4.11374 40290244 PMC12022588

[59] Li Z, Zhao D, Zhu C. Predicting colorectal adenoma recurrence: the role of systemic inflammatory markers and insulin resistance. Scand J Gastroenterol. 2025;60(4):300–6. doi: 10.1080/00365521.2025.2469801 40009759

