# Supplementary material for: Combined predictive value of TyG index and PLR for mental health in Chinese adults: A machine learning approach
Source: PLoS One. 2026 Sep 2;21(9):e0355937. doi: 10.1371/journal.pone.0355937 (PMC13537578; doi:10.1371/journal.pone.0355937)
Supplement: Supplementary file 1 [file pone.0355937.s002.docx]

**Table S1 Summary of dataset variables, types, coding, and roles in analysis**

| **Variable** | **Type** | **Unit / Coding** | **Role** |
| --- | --- | --- | --- |
| Age | Continuous | Years | Covariate |
| Gender | Categorical | 1: Male; 2: Female | Covariate |
| BMI | Categorical | 1: Underweight; 2: Normal; 3: Overweight; 4: Obesity | Covariate |
| Smoking status | Categorical | 0: Yes; 1: No | Covariate |
| Alcohol consumption | Categorical | 0: Yes; 1: No | Covariate |
| Education level | Categorical | 1:≤Junior high; 2:High school; 3:College; 4:degree; ≥College | Covariate |
| Diabetes | Categorical | 1: Yes; 0: No | Covariate |
| Hypertension | Categorical | 1: Yes; 0: No | Covariate |
| Dyslipidemia | Categorical | 1: Yes; 0: No | Covariate |
| PLR | Continuous | — | Dependent Variable |
| TyG Index | Continuous | — | Dependent Variable |
| SCL-90 total score | Continuous | — | Independent Variable |
| Mental health | Categorical | 1: Yes; 0: No | Independent Variable |

**Table S2 Parameters of machine learning models**

| Model | Best-tuned Parameters |
| --- | --- |
| LASSO | λ = 0.007476 |
| Logistic Regression | No hyperparameter tuning |
| XGBoost | eta = **0.05**  max_depth = **3**  min_child_weight = **3**  subsample = **0.8**  colsample_bytree = **0.8**  lambda = **1**  scale_pos_weight = **1**  best_nrounds = **(cv_result$best_iteration)** |
| Random Forest | mtry = **2**  ntree = **1000** |
| SVM | gamma = **0.1**  cost = **1** |
| GBM | n.trees = **300**  interaction.depth = **3**  shrinkage = **0.1**  n.minobsinnode = **10** |
| Neural Network | hidden = **c(8, 4)**  activation = **logistic** (sigmoid)  linear.output = **FALSE**  threshold = **0.1**  stepmax = **1e6** |
